# Structural motif screening reveals a novel, conserved carbohydrate-binding surface in the pathogenesis-related protein PR-5d

**DOI:** 10.1186/1472-6807-10-23

**Published:** 2010-08-03

**Authors:** Andrew C Doxey, Zhenyu Cheng, Barbara A Moffatt, Brendan J McConkey

**Affiliations:** 1 Department of Biology, University of Waterloo, 200 University Avenue West, Waterloo, Ontario, N2L 3G1, Canada; 2 Department of Developmental Biology, Stanford University, Stanford, CA, 94305, USA

## Abstract

**Background:**

Aromatic amino acids play a critical role in protein-glycan interactions. Clusters of surface aromatic residues and their features may therefore be useful in distinguishing glycan-binding sites as well as predicting novel glycan-binding proteins. In this work, a structural bioinformatics approach was used to screen the Protein Data Bank (PDB) for coplanar aromatic motifs similar to those found in known glycan-binding proteins.

**Results:**

The proteins identified in the screen were significantly associated with carbohydrate-related functions according to gene ontology (GO) enrichment analysis, and predicted motifs were found frequently within novel folds and glycan-binding sites not included in the training set. In addition to numerous binding sites predicted in structural genomics proteins of unknown function, one novel prediction was a surface motif (W34/W36/W192) in the tobacco pathogenesis-related protein, PR-5d. Phylogenetic analysis revealed that the surface motif is exclusive to a subfamily of PR-5 proteins from the Solanaceae family of plants, and is absent completely in more distant homologs. To confirm PR-5d's insoluble-polysaccharide binding activity, a cellulose-pulldown assay of tobacco proteins was performed and PR-5d was identified in the cellulose-binding fraction by mass spectrometry.

**Conclusions:**

Based on the combined results, we propose that the putative binding site in PR-5d may be an evolutionary adaptation of Solanaceae plants including potato, tomato, and tobacco, towards defense against cellulose-containing pathogens such as species of the deadly oomycete genus, *Phytophthora*. More generally, the results demonstrate that coplanar aromatic clusters on protein surfaces are a structural signature of glycan-binding proteins, and can be used to computationally predict novel glycan-binding proteins from 3 D structure.

## Background

Carbohydrate-binding proteins (CBPs) are highly diverse in terms of their sequences, structures, binding sites, and evolutionary histories [1]. Sequence-based classifications (e.g., as used in the CAZy database [2]) are an attempt to organize this diversity, and do so by grouping CBPs into evolutionarily related families and subfamilies. Many of these families have a common function and mechanism, while in others functions have diversified [2]. Prediction of novel CBPs with unique binding sites and mechanisms that are unrelated to known cases is a more difficult task, as there is no single sequence profile or pattern that defines a carbohydrate-binding site. Thus, while sequence-based carbohydrate-binding site prediction methods have been shown to be moderately successful, structural information will be key to achieve higher prediction accuracies [3].

Structure-based algorithms are a promising approach for prediction and analysis of binding sites in proteins from first principles. Just as sequence profiles and patterns can be used to infer function in uncharacterized sequences, the existence of specific structural patterns in incompletely characterized structures may provide clues into their functions [4,5]. As binding site residues and other functional motifs may be close in 3 D space but be non-contiguous in the amino acid sequence, structural patterns are inherently better at representing proteins functions than primary sequence alone. A number of structure-based approaches have been applied to carbohydrate-binding site prediction, and have achieved reasonable prediction accuracy [6-8]. However, even using structural information, not all carbohydrate-binding sites can be correctly predicted (e.g., false negative rates are roughly 30%). Structure-based prediction of CBPs with novel folds and binding sites has also not been performed and validated experimentally. Given their enormous potential in biotechnological applications [9], computational prediction of novel CBPs is a worthwhile goal.

It is unlikely that general feature-detection approaches will be able to identify all types of carbohydrate-binding sites. Carbohydrate ligands are diverse in size, geometry and other physicochemical characteristics [2], and this diversity is mirrored in the features of carbohydrate-binding sites in proteins. A few recent studies have developed more targeted approaches that apply structure-based methods to specific classes of CBPs [10,11]. At a cost of lower generality, approaches that focus on structural motifs of particular functional classes of CBPs may achieve predictions with better ligand specificities and greater overall accuracies.

A useful structural and functional classification of CBPs is described by Boraston et al. [1]. Carbohydrate-binding modules (CBMs) were divided into three main types (type A, B and C) based on their structural and functional characteristics, where members of each class are not necessarily related and do not share a common sequence pattern. Type A CBMs, which bind insoluble carbohydrates, possess a unique structural signature of three surface aromatic residues whose side-chains are arranged in a coplanar orientation to dock to a crystalline carbohydrate surface. In the binding sites of type B (glycan-chain binding) CBMs, there are typically two coplanar aromatic residues which form a "sandwich" or "clamp" around the glycan ligand. Through hydrophobic stacking (CH-Π) interactions [12,13], aromatic sidechains of Type A and B CBMs bind to their respective glycan ligands, which are polysaccharides or oligosaccharides. Smaller monosaccharides, however, are the targets of type C CBMs, which do not necessarily possess coplanar aromatic motifs. The use of aromatic motifs as structural signatures of CBPs is consistent with computational and experimental analyses of carbohydrate-binding sites. Malik et al. [3], who scored amino acid propensities in known carbohydrate-binding sites, found that Trp is extremely overrepresented (331%). Mutations of aromatic residues such as Trp have also been shown to significantly decrease carbohydrate-binding activities [14-17]. Ultimately, these studies indicate that Trp and Tyr are highly prevalent in carbohydrate-binding sites, while Phe and His are found less frequently. The abundance of Trp is partially due to it having the largest surface area of all amino acids for potential hydrophobic interactions. The relative abundance of Tyr over Phe is explained by the ability of Tyr to form additional H-bonds and electrostatic interactions because of its hydroxyl group. Thus, even within aromatic residues, subtle sidechain differences have the potential to affect carbohydrate-recognition.

While binding sites like those found in type C CBMs may be more structurally diverse and thus harder to identify using a structural signature, aromatic motifs found in type A and B CBM binding have the potential to be used as 3 D motifs in structural database screening to identify novel carbohydrate-binding sites [10]. In this work, we expand on a previous 3D-motif approach [10] to perform a comprehensive PDB-wide screen for coplanar aromatic surface motifs. The primary goal is to determine whether such motifs are significantly enriched in carbohydrate-related proteins and can be used to identify novel CBPs and binding sites not found in existing CBP families. A novel prediction (pathogenesis-related protein, PR-5d) is then analyzed computationally and tested experimentally.

PR-5d refers to the tobacco pathogenesis-related protein, which is a member of the larger PR-5 family, including the proteins thaumatin and osmotin. Anti-fungal activity has been demonstrated for PR-5d [18] and related PR-5 proteins [19,20], but the structural basis of this activity is still unclear. Membrane pore-formation has been suggested as one possible anti-fungal mechanism [18]. Carbohydrate-binding and hydrolytic functions have also been observed for a number of PR-5 proteins (e.g., ß-1, 3-glucan interactions in thaumatin-like proteins [21,22]). Carbohydrate interactions are consistent with structural modelling studies of PR-5 proteins, which have demonstrated that PR-5 proteins contain highly acidic clefts suitable for carbohydrate hydrolytic function [22,23]. This suggests that the mechanism may involve interactions between PR- 5 proteins and pathogen cell wall carbohydrates. The PR-5d surface motif predicted in this study is therefore of particular interest because pathogen-specific insoluble-carbohydrate binding may represent a previously unknown mechanism by which PR-5d acts on specific pathogens of tobacco and related species. Furthermore, the results of this work highlight a critical surface region on which subtle mutations may underlie important functional novelties in the PR-5 family. In a broader sense, this study highlights the potential of structural motif screening approaches to predict novel functions using large-scale structural data.

## Results and Discussion

### Linear discriminant analysis of coplanar aromatic surface motifs

**Figure 1:**
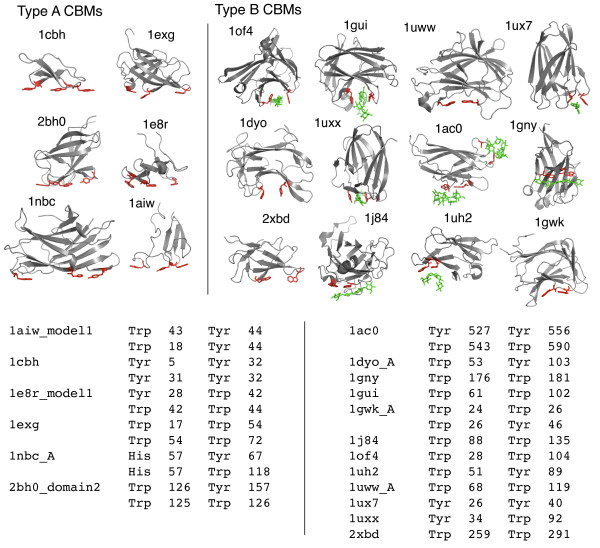
**Aromatic surface motifs in a training set of type A and B CBMs**. Structures of 6 type A and 12 type B CBMs and their glycan-binding aromatic motifs (highlighted in red) and ligands (highlighted in green if present in the PDB structure). PDB ids and aromatic residue pairs used in training are listed for each structure.

To determine whether coplanar aromatic surface motifs like those found in type A and B CBMs are structural signatures of glycan-binding proteins, linear discriminant analysis (LDA) was applied to a training set of coplanar aromatic motifs occurring in structures of known glycan-binding proteins. Positive cases used in training included 26 pairs of glycan-binding aromatic residues in known type A and B CBM binding sites from 18 different structures (Figure 1). Negative cases used in training included 140,383 random pairs from the Nh3d reference dataset [24], further filtered to 7,830 pairs by selecting only those whose features were no worse than the worst-case values found in the positive cases (see thresholding section in Methods). This conservative approach is intended to identify and separate putative carbohydrate binding motifs from similar inactive aromatic groups on protein surfaces.

**Figure 2:**
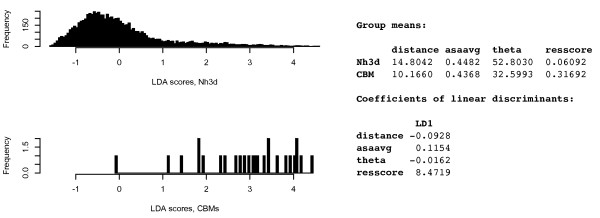
**LDA-based discrimination of selected aromatic pair motifs in known type A and B CBMs, and reference aromatic pairs from the Nh3d dataset passing the initial thresholds**. Four features (distance, ASA, coplanarity, and residue type) were used in the discrimination. Parameter means and LDA statistics are listed on the right.

Based on coplanarity of aromatic sidechains, solvent accessibility, residue type, and distance, LDA was able to effectively separate the two classes based on the input features (Figure 2). Twenty out of 26 (77%) of the positive cases scored greater than 95% of background scores, and 24 out of 26 (92%) scored greater than 90% of the background scores.

The coefficients of linear discriminants, which provide an indication of the contribution and relationship of each variable to the discrimination, are shown in Figure 2. The signs of each coefficient are as expected; the distance parameter is negative indicating a preference for aromatic pairs in closer proximity; solvent accessibility (asaavg) is positive indicating a preference for greater surface exposure; and theta is negative indicating that lower angles (higher coplanarity) is preferred. The residue-type parameter resscore was the strongest discriminating variable between aromatic motifs found in glycan-binding sites and random, aromatic surface motifs.

### PDB screening reveals a significant association between aromatic surface motifs and carbohydrate related functions

After parameter fitting, the algorithm was applied to 15,970 non-redundant structures from the nrPDB dataset, with an initial 229,919 aromatic pairs from 15,047 different structures that passed the parameter thresholds. These were screened for pairs that received a raw LDA score greater than the 99th percentile score (~3.40) of all sites analyzed in the initial training set. This resulted in 1,304 high-scoring aromatic pairs from a total of 994 unique structures.

To determine whether the proteins identified by screening are enriched in carbohydrate-related functions, GO terms for all structures were retrieved using the GOA database [25], and GO terms for proteins identified in the screen (which excluded structures from the training set) were assessed for statistical enrichment using the binomial test (see Methods). Out of 501 total GO associations for proteins in the set of 994 structures identified in the screen, 14 significantly enriched GO terms were detected according to the binomial test with a false-discovery rate (FDR) adjustment for multiple testing [26] and conservative FDR cutoff of 0.05 (Table 1). Nine of these are associated with carbohydrate-related functions (Table 1). For example, out of 380 structures in the nrPDB annotated with GO: 0005975 ("carbohydrate metabolic process"), 95 of these were identified in the screen. Thus, a quarter of the structures with this annotation contain an aromatic pair motif that scores higher than the 99^th ^percentile. Of 48 structures in the nrPDB that contain the term GO: 0030246 (carbohydrate-binding), 17 of these were identified. This is equivalent to a four-fold enrichment (p ~ 1.57e-29), and over five-fold enrichment (p ~ 1.9e-08) in carbohydrate metabolism and binding functions, respectively. When the screening threshold score is lowered, a larger proportion of structures from these GO categories are identified, but at the expense of more false positives. For example, 40, 31, and 24 of the 48 carbohydrate-binding structures were identified when searching for motifs scoring greater than the 75^th^, 90^th^, and 95^th ^percentile scores, respectively. This demonstrates that many carbohydrate-binding proteins not identified in the top 99^th ^percentile screen also possess similar aromatic motifs that are simply lower-scoring.

**Table 1:** Enriched GO functions in structures identified by motif screening.

GO TERM	GO description	# in screen	# in dataset	Enrichment	P (raw)	FDR cutoff
GO: 0004553	hydrolase activity, hydrolyzing Oglycosyl compounds	71	171	6.67	4.26E-35	9.98E-05
GO: 0005975	carbohydrate metabolic process	95	380	4.02	1.57E-29	2.00E-04
GO: 0043169	cation binding	50	145	5.54	1.22E-21	2.99E-04
GO: 0004568	chitinase activity	13	18	11.60	2.33E-10	3.99E-04
GO: 0006032	chitin catabolic process	12	17	11.34	1.47E-09	4.99E-04
GO: 0030246	carbohydrate binding	17	48	5.69	1.86E-08	5.99E-04
GO: 0008810	cellulase activity	8	14	9.18	3.73E-06	6.99E-04
GO: 0003824	catalytic activity	139	1622	1.38	8.24E-05	7.98E-04
GO: 0030245	cellulose catabolic process	6	13	7.42	1.94E-04	8.98E-04
GO: 0006662	glycerol ether metabolic process	6	13	7.42	1.94E-04	9.98E-04
GO: 0008061	chitin binding	4	5	12.85	3.04E-04	1.10E-03
GO: 0000272	polysaccharide catabolic process	7	22	5.11	5.40E-04	1.20E-03
GO: 0015343	siderophore-iron transmembrane transporter activity	3	3	16.07	9.41E-04	1.30E-03
GO: 0015891	transport	3	3	16.07	9.41E-04	1.40E-03

For some apparently enriched terms not directly related to carbohydrate-binding (e.g., 'cation-binding'), these terms are commonly linked with carbohydrate-related enzymes and thus exhibit significant enrichment in the dataset. For example, 1LWJ (*T. Maritima *4-alpha-glucanotransferase/acarbose complex), is tagged with GO: 0005975 (carbohydrate metabolic process) as well as GO: 0003824 (catalytic activity), and GO: 0043169 (cation binding). All three of these terms showed significant enrichment (Table 1).

### Example predictions

**Figure 3:**
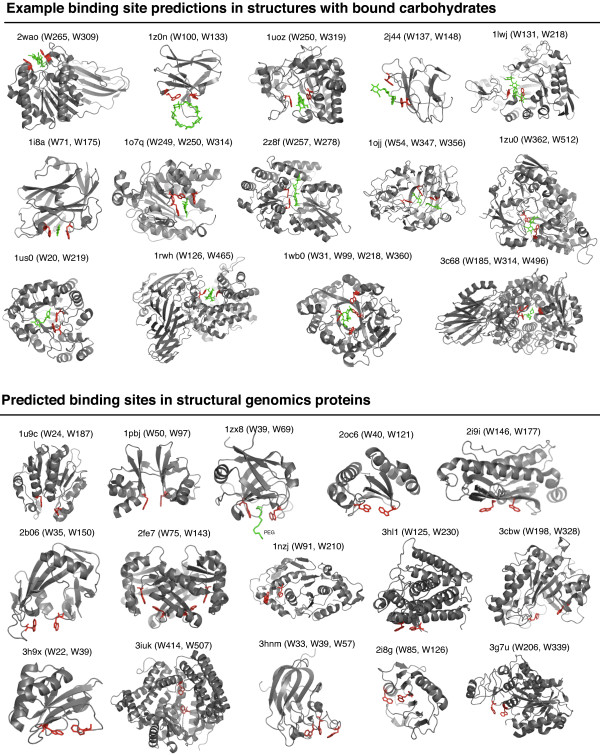
**Selected binding site predictions obtained by PDB screening**. Correctly identified binding motifs found in known glycan-binding proteins (top panel); novel predictions occurring in structural genomics proteins whose functions are unknown or incompletely characterized (bottom panel).

Representative examples of several correctly identified glycan-binding sites in structures not included in the training set are shown in Figure 3 (top panel). In the selected structures, predicted aromatic motifs with raw LDA scores > 99^th ^percentile have been highlighted along with their corresponding bound glycan. Each predicted binding site and most of the folds are unique, and the aromatic motifs have in these cases have arisen independently through convergent evolution.

Also shown are 17 predicted aromatic-motif binding sites occurring in structural genomics proteins whose functions are incompletely characterized or unknown (Figure 3, bottom panel). While carbohydrates/glycans are likely targets of the predicted motifs, they may also be binding sites for other ligands (e.g., some nucleotide-binding sites also consist of an aromatic sandwich motif).

The detected motifs are similar to those found in type A and B CBMs, and are predominantly Trp-rich coplanar strip or sandwich motifs. All examples shown in Figure 3 contain Trp/Trp motifs, as this was the most highly scored residue combination and thus occurs most frequently in the top percentile of scoring. All of the structural genomics proteins shown have different folds from the structures in the training set, with one exception. PDB ID 3hnm (a domain from a putative chitobiase) structurally aligned well with the CBM 22 xylan-binding domain (1dyo) according to VAST (E-value = 0.0453) [27], but has very a low sequence identity (5.2%) to this protein.

In several of these cases, sequence or structural similarities also support the possibility of carbohydrate-binding functions. PDB ID 1u9c is in the same SCOP superfamily ('Class I glutamine amidotransferase-like') as A4 beta-galactosidase middle domain (PDB ID 1kwk). A BLAST search of 2i9i detected similarity to "neuraminyllactose-binding hemagglutinin" proteins (e.g., PDB ID 3bgh). PDB ID 2b06 has a nudix fold also found in GDP-mannose mannosyl hydrolase NudD (e.g., PDB ID 1rya). PDB ID 1pbj has a CBS-domain pair fold, and a similar site in PDB ID 2rif binds AMP. Lastly, 3cbw is a structure of beta-mannanase BsMan26A from *Bacillus subtilis *[28].

The motifs shown in Figure 3 were identified using an LDA score threshold equivalent to the 99^th ^percentile score. However, it is important to note that other known and candidate carbohydrate-binding structures had motifs scoring below this threshold. For example, the structural genomics proteins, PDB ID 3e5z (a putative gluconolactonase; predicted residues: W29, Y135) and PDB ID 3dsm (*B. uniformis *surface layer protein; predicted residues: W117, W162, Y207, W240, Y284, Y303) are likely to interact with carbohydrates and had predicted binding sites scoring highly (>95% score) but below the threshold.

### A high-scoring aromatic motif on the surface of pathogenesis-related protein, PR-5d

One of the top predictions was a surface motif in the pathogenesis-related protein PR-5d from tobacco. The putative binding site in PR-5d received a raw LDA score of ~3.80 (99.6^th ^percentile) for the W34/W36 pair, ~3.69 (99.5^th ^percentile) for the W36/W196 pair, and ~3.15 (98.6^th ^percentile) for the W34/W196 pair. Thus, the motifs in PR-5d received extremely high scores, scoring higher than many of the glycan-binding sites from the training set. Compared to all proteins in the nrPDB analyzed in screening, this putative binding site had scores greater than 99.7% of cases.

**Figure 4:**
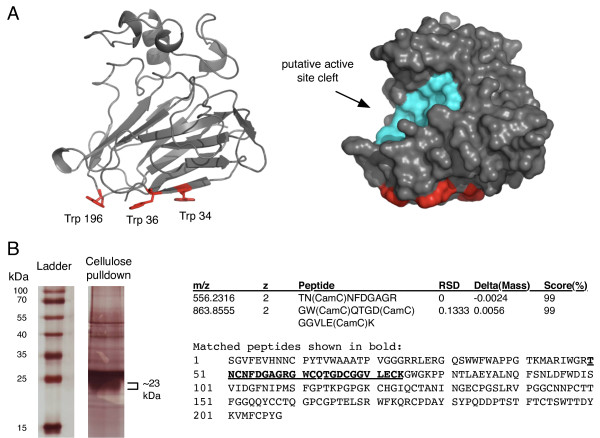
**A predicted binding-site in PR-5d and experimental validation of PR-5d insoluble-carbohydrate binding activity using a cellulose pulldown assay and mass spectrometry**. (A) The predicted Trp-rich binding surface in tobacco antifungal protein, PR-5d (PDB ID 1aun). A surface representation is also shown to illustrate the hydrolytic cleft region, which is distinct from the predicted binding site. (B) A silver stained gel of purified cellulose-binding proteins from tobacco (left). The marked band was excised from the corresponding position on a coomassie stained gel and identified by mass spectrometry (MS). MS sequencing identified two peptides matching the PR-5d sequence. MS statistics and sequence information is shown to the right of the gel, where CamC represents carbamidomethyl derivatives of cysteine residues.

The crystal structure of tobacco PR-5d (PDB ID 1aun) is shown in Figure 4A. The predicted binding site in PR-5d comprises three Trp residues (W34, W36, W196), which form a highly coplanar and accessible surface region reminiscent of coplanar aromatic surface motifs found in the binding sites of type A CBMs (Figure 1). It forms a separate and distinct surface patch from the putative active site cleft (Figure 4A).

In addition to PR-5d, only 51 other structures out of 15,970 (< 0.3%) were found to contain type A CBM-like triplets with scores greater or equal to that of PR-5d. Such binding sites composed of three or more coplanar aromatic motifs were identified by grouping together pairs of high-scoring coplanar aromatics that share a common residue.

Given previous associations between the hydrolytic cleft and glycan binding [21-23], it is possible that motifs within or near the hydrolytic cleft of PR-5d also contribute to cellulose binding. In addition to W34/W36/W196, a weaker scoring pair was detected near the hydrolytic cleft (F91 and F96). This site received an LDA score of 0.505, placing it in the 86th percentile of all scores. Though this site and other motifs in the hydrolytic cleft are suitable for binding glycan chains, they do not possess the common pattern of type A CBM binding sites, and are much less likely to be capable of binding to a crystalline-cellulose surface.

### An insoluble cellulose-pulldown assay of tobacco proteins identifies PR-5d

The structural analysis indicates that PR-5d possesses a significant structural signature of carbohydrate-binding proteins, the W34/W36/W196 motif that is similar to insoluble-carbohydrate binding motifs found in known type A CBMs. Thus, the binding activity of PR-5d towards insoluble cellulose was tested experimentally using a cellulose pulldown assay of tobacco (*Nicotiana tabacam*) proteins, followed by mass spectrometry. A similar experiment performed with insoluble chitin was also performed in a previous study, which identified a chitinase CBM but not PR-5d [10]. Tobacco plants were first treated with salt in order to cause an ethylene-induced stress response in order to induce PR-5d gene expression (previously shown in Sato et al. [29]) and obtain a larger PR-5d yield than that expected under normal conditions. In the cellulose-pulldown assay, tobacco protein extract was mixed with insoluble cellulose, and the mixture was washed stringently in order to remove non-cellulose-binding proteins and purify only proteins with strong binding activity towards insoluble cellulose. The cellulose-binding fraction was then analyzed with SDS-PAGE, stained with both coomassie and silver stain, and analyzed by mass spectrometry. The stringent conditions resulted in several faint bands on the coomassie stained gel, which were more easily visualized using silver stain (Figure 4B). A band at the expected size (~23 kDa) of PR-5d was present, which was then excised and identified by mass spectrometry (MS). MS analysis identified two peptides, which were identified as the top-scoring match to the sequence of the tobacco PR-5d protein (Figure 4B). The identification of PR-5d in the cellulose-binding fraction demonstrates that PR-5d has insoluble-cellulose-binding activity and validates the computational prediction.

### Phylogenetic analysis of the PR-5d W34/W36/W196 motif

To further support the functional importance of the cellulose-binding motif, patterns of residue conservation across species were investigated. Close homologs of PR-5d were retrieved via a BLAST search of the NCBI nr protein database and a multiple alignment and phylogenetic tree was constructed. The predicted aromatic motif residues and corresponding residues from other related PR-5 proteins were then mapped onto the phylogeny in order to phylogenetically trace the origin of the W34/W36/W196 motif in PR-5d.

**Figure 5:**
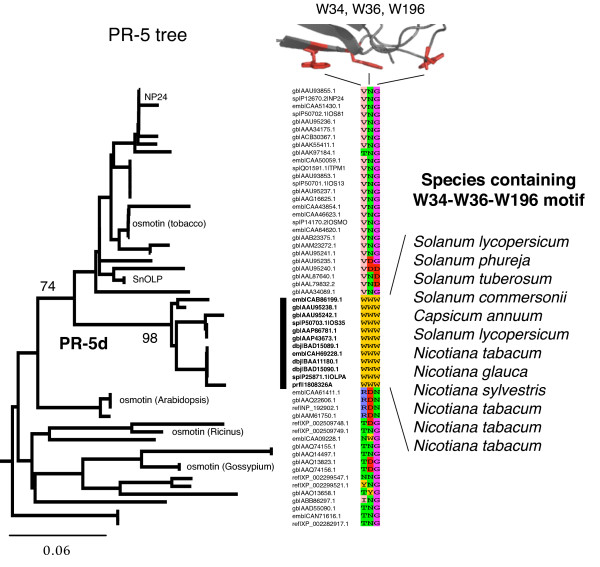
**Phylogenetic analysis of PR-5d and the W34/W36/W196 aromatic surface motif**. The tree of PR-5d and related proteins is a subtree from a larger neighbor-joining tree of PR-5 related proteins. Bootstrap values are indicated for two key clades. The residues in alignment positions 34, 36, and 196 (relative to the PR-5d sequence) have been mapped onto the tree, and demonstrate that the conserved putative binding site (W34/W36/W196) likely originated in an ancestral PR-5d protein in an ancestral species of the Solanaeceae family. The scale bar indicates units of number of amino acid substitutions per site.

Phylogenetic analysis revealed that PR-5d is a member of a highly conserved clade of PR-5 proteins exclusive to the Solanaceae family of plants including tomato (*Solanum lycopersicum*), potato (*S. tuberosum*), chili pepper (*Capsicum annuum*), and several species of tobacco (*N. tabacum*) (Figure 5). This clade of PR-5d proteins, extremely well supported by a bootstrap value of 98/100, all share the W34/W36/W196 motif, which is not present anywhere else in the phylogeny (Figure 5). The 100% conservation indicates the functional importance of this clade-defining motif, and suggests it may be a potential evolutionary and functional determinant of this group of Solanaceae PR-5 proteins.

### A proposed function of the Solanaceae specific PR-5d subfamily

Solanaceae plants are common targets of the deadly plant pathogen, *Phytophthora *(Greek for "plant-destroyer"). The clade-defining, putative binding site towards insoluble-polysaccharides in the plant-defense protein, PR-5d, may be an evolutionary adaptation towards defense against *Phytophthora*. Unlike fungi, which are commonly studied targets of PR-5 proteins, a distinguishing feature of *Phytophthora infestans *and other oomycetes is that they possess a cell wall containing insoluble cellulose [30,31]. Previous studies have shown that PR-5 related proteins such as tobacco osmotin are active against *Phytophthora infestans *[19,32]. According to the tree, tobacco osmotin and related osmotin-like proteins are indeed the most closely related sequences to the PR-5d subfamily (several of these proteins are labelled in Figure 5). The occurrence of the W34/W36/W196 motif may therefore represent a functional innovation in response to *Phytophthora *invasion and infection, providing additional indirect support for the mechanistic importance of this motif.

## Conclusion

Through this work, it has been shown that key features of surface aromatic motifs (residue type, distance, solvent-accessibility, and coplanarity) can be used to structurally distinguish known and novel glycan-binding proteins and their binding sites from random protein structures. This provides further support for approaches that use physicochemical and geometric features of protein surfaces to analyze and predict specific protein functions [33-35]. By performing a large-scale screen of the PDB using the 3 D pattern, existing carbohydrate-binding sites were correctly predicted as well as novel motifs in structural genomics proteins of unknown function. In addition, aromatic surface motifs were found to be significantly associated with carbohydrate-related functions. A high-scoring prediction (PR-5d) was studied structurally, phylogenetically and experimentally. The combined results suggest that the W34/W36/W196 surface motif in PR-5d may function as an insoluble-polysaccharide binding site that targets polysaccharides such as cellulose in pathogen cell walls. A likely target is the major plant pathogen, *Phytophthora*, which has a cell wall made of cellulose, and which commonly infects the Solanaceae species in which the PR-5d W34/W36/W196 motif is present.

## Methods

### Structural dataset construction

Type A and B CBMs: Representative structures of six type A CBMs and 12 type B CBMs were retrieved from the Protein Data Bank (PDB), yielding a total of 17 diverse carbohydrate-binding structures. All structures have distinct folds or low pairwise sequence identity (BLASTp E-values greater than 0.01). Type A CBMs included 1cbh (CBM1), 1exg (CBM2), 1nbc (CBM3), 1aiw (CBM5), 1e8r (CBM10), and 2bh0, a recently solved structure of a bacterial expansin with similarity to plant beta-expansins (group 1 grass pollen allergens). This structure also has a binding site and activity analogous to type A CBMs [36]. Type B CBMs included 2xbd (CBM2), 1gui (CBM4), 1uxx (CBM6), 1gny (CBM15), 1j84 (CBM17), 1ac0 (CBM20), 1dyo (CBM22), 1of4 (CBM27), 1uww (CBM28), 1gwk (CBM29), 1uh2 (CBM34), and 1ux7 (CBM36). These structures were selected based on the scheme presented in Boraston et al. [1], a comprehensive review on CBMs.

Nh3d reference dataset: The Nh3d version 3.0 dataset [24] was retrieved for use as the reference dataset. This dataset contains 806 structurally dissimilar protein chains from the PDB, and was built using the hierarchical CATH database classification. Nh3d was chosen because it was carefully constructed to contain structurally dissimilar protein chains without recognizable common ancestry, and so it lends itself to statistical, structural analysis. In addition, none of the proteins in this dataset are closely related (as determined through BLAST searches) to the sequences in the type A and B CBM dataset. nrPDB: For the purposes of screening, a large non-redundant database of 15,970 PDB structures was generated by retrieving a precomputed list of structures from the PISCES server [37]. The percentage identity cutoff was 90%, the resolution cutoff was 3.0 Å, and all R-factors were allowed. Homologs of the type A and B CBM structures were not included in this dataset.

### Structural motif analysis and screening

Aromatic residue pairs were selected within known structures from the type A and B CBM dataset based on previous literature and manual inspection (aromatic pairs listed in Figure 1). For type B CBMs, one pair of aromatic residues was used to describe a single ligand-binding site, and for type A CBMs composed of three aromatic residues, the two pairs with the shortest inter-residue distance were used. As a comparison reference dataset containing random protein structures, the Nh3D dataset was used. All aromatic pairs passing initial parameter thresholds (identical to those later described in *Screening*) were selected, and assumed as non-binding-sites for algorithm training.

Four key features (coplanarity, residue type, solvent-accessibility, and distance) were computed for all pairs of aromatic residues (Trp, Tyr, Phe, His) and used in a linear discriminant analysis (LDA) in an attempt to discriminate the known sites from the random sites (background). LDA generated a scoring function based on a linear combination of the input variables, that best separates the two classes of data. The linear discriminant function can be represented as:

Here, Z_ip _are the values of the discriminating variables; B_p _are the coefficients; and D_i _is the score for the i^th ^data point (in this case, putative binding site). We used the raw LDA score in subsequent searches for novel binding sites. The following features were used to train the parameters in LDA:

Coplanarity: measured as the angle (0 to 90 degrees) between the normal vectors of both aromatic rings).

Residue type: The score of each residue pair was set to the frequency of the pair plus a pseudocount of 0.5 (except for Phe because Phe was not observed at all). The scores for each residue pair were divided by the sum of total scores, resulting in (Trp/Trp = 0.431; Trp/Tyr = 0.293; Tyr/Tyr = 0.155; Trp/His = 0.052; His/His = 0.017; Any/Phe = 0).

Distance: the Euclidean distance, sqrt[(x_2_-x_1_)^2^+(y_2_-y_1_)^2^+(z_2_-z_1_)^2^], between the centroids [(x_1_,y_1_,z_1_) and (x_2_,y_2_,z_2_)] of each aromatic ring.

Solvent accessibility: the solvent accessible surface area (ASA) was calculated from a Voronoi tessellation [38]. To account for differences in inherent sidechain solvent accessibility between different aromatic residues, each value was divided by the maximum observed in the background dataset for that residue. The average relative ASA value of the two aromatic sidechains was then used as a final feature.

*Screening*: In the screening phase, a separate database of 15,970 non-redundant structures (nrPDB) was screened for potentially novel binding glycan-binding sites and other binding-sites not included in the training set. This involved two steps:

1) Thresholding: aromatic pairs with feature values outside the allowed range were removed. The allowed range was simply defined based on the minimum and/or maximum values observed for known binding sites in the training set (6.03 Å ≤ Distance ≤ 21.03 Å, Fractional solvent-accessibility relative to residue type ≥ 0.21, Coplanarity ≤ 83.55 degrees). The ASA cutoff, for instance, removed internal aromatic residues incapable of forming external interactions.

2) Scoring: The LDA scoring function was used to score all remaining candidate binding sites. The score of any aromatic pair can be compared to the "background" distribution of scores, which reflects its potential for being a glycan- or other type of ligand-binding site.

### Gene Ontology (GO) analysis

PDB GO annotations were downloaded from the Gene Ontology Annotation (GOA) database of the European Bioinformatics Institute http://www.ebi.ac.uk/GOA. For structures identified by screening, GO term enrichment was tested for all GO categories associated with the identified structures. Binomial exact tests were used to compute the probabilities *P_k _*of observing *k *or more instances of a particular GO term in the screen (*n *= 994). Assuming that the background probability *p *of observing a particular GO term is (total # occurrences/total # structures), the probability *P_k _*is:

Since this test was done for all *N *= 501 GO terms, we used false-discovery rates to correct for multiple statistical tests. The 501 P-values were first ranked in increasing order, and significant p-values were those for which the raw p-value is less than (rank × alpha/*N*) [26]. An FDR alpha value of 0.05 was used. The fold enrichment (fraction of GO term observed in the screen/fraction of GO terms in all structures) is also reported in Table 1.

### Cellulose pulldown assay

Ten grams of root tissues from 3-week old tobacco plants were ground with liquid nitrogen and homogenized with 10 mL of extraction buffer (20 mM HEPES pH 8.0, 0.5 M NaCl, 0.1 mM EDTA pH 8.0, 0.1% Triton-X100). The suspension was sonicated 3 × 30 seconds with 30-second pauses between pulses at 200-300 W. The cell lysate was centrifuged at 10000 × *g *at 4°C for 10 minutes. A protein concentration of 1 μg/μL was determined using the Bradford assay. 0.5 grams of Avicel® microcrystalline cellulose (~1 mL) that was purified from fibrous plants (FMC Corporation, Newark, DE) was equilibrated with 10 mL of extraction buffer. The whole 10 mL of lysate was then mixed with the equilibrated cellulose and incubated at 4°C for 3 hours. The mixture was applied to a Poly-Prep^® ^chromatography column (Bio-Rad Laboratories, Hercules, CA). Loosely bound proteins were removed by washing with 30 mL (~30 column volumes) of extraction buffer. The cellulose-binding proteins were eluted with 4 × 1 mL of 0.1% SDS, and the eluate was collected as the cellulose-binding fraction.

The cellulose-binding fraction was separated by 12% SDS-PAGE gel and stained with Coomassie blue G-250 (Bio-Rad Laboratories, Hercules, CA). The strongest bands were excised and digested with trypsin as previously described [39]. The peptides were extracted from gel pieces by vortexing and dried in a SpeedVac (Instruments Inc., Hicksville, NY). The peptides were resuspended in 50% acetonitrile with 0.1% formic acid. Mass spectrometry was performed on an Applied Biosystems Q-TRAP system. Peak lists were generated and processed using Analyst software version 1.4.1 (Applied Biosystems). The protein was identified using PEAKS version 4.5 (Bioinformatics Solutions Inc., Waterloo, ON). The parental and fragment mass error are 0.2 Da and 0.1 Da, respectively. Fragments were predicted based on digestion with trypsin (one missed cleavage site allowed) and carbamidomethylation and methionine oxidation as fixed and variable modifications, respectively.

As the Coomassie gel produced faint bands, a second SDS-PAGE gel was prepared and silver stained. The silver staining procedure was followed by the instruction of the PlusOne Protein Silver Staining Kit from GE Healthcare (cat # 17-1150-01).

### Phylogenetic and sequence analysis of PR-5d

A BLAST search of tobacco PR-5d (PDB ID 1aun) was used to identify related sequences. All sequences with E < 0.001 that aligned to the query with sequence coverage > 90% were used to build a second alignment using MUSCLE [40]. Conserved regions of the alignment were used to generate a midpoint-rooted neighbor-joining tree using Seaview [41]. A major clade of PR-5d proteins containing the PR-5d subclade was then selected for further analysis.

## Authors' contributions

ACD and BJM conceived and designed the study, and ACD carried out the computational analyses. BAM assisted with design of experiments and techniques for isolation of plant proteins. ZC performed the affinity purification assay and mass spectrometry analysis. All authors read and approved the final manuscript.
